# The Challenge of Confronting Ageism: Impressions of Targets and Bystanders Who Intervene

**DOI:** 10.1093/geroni/igaa057.2225

**Published:** 2020-12-16

**Authors:** Michelle Horhota, Alison Chasteen, Monika Schindwolf

**Affiliations:** 1 Furman University, Greenville, South Carolina, United States; 2 University of Toronto, Toronto, Ontario, Canada

## Abstract

Does confronting ageism come with a cost? Benevolent ageism is viewed as more appropriate than hostile ageism which may lead to negative consequences for individuals who confront it. We examined whether impression costs are mitigated or exacerbated by the style of confrontation (moderate or strong) and the person who confronts (the target or a bystander). Young and older participants read a vignette and rated the target, perpetrator and bystander on warmth, competence, and the acceptability of each character’s actions. Participants rated targets who confronted more negatively than bystanders who confronted, and preferred moderate over strong confrontation. In addition, participants thought the perpetrator would be less likely to exhibit prejudicial behaviors again if the older target confronted the action rather than the bystander. This demonstrates the challenge that older adults face; confronting results in a negative impression of them but may be more effective in preventing ageist actions in the future.

